# Japan’s Drug Regulation Framework: Aiming for Better Health or Bigger Profits?

**DOI:** 10.34172/ijhpm.2020.56

**Published:** 2020-04-19

**Authors:** Akihiko Ozaki, Yuki Senoo, Hiroaki Saito, Andy Crump, Tetsuya Tanimoto

**Affiliations:** ^1^Department of Breast Surgery, Jyoban Hospital of Tokiwa Foundation, Fukushima, Japan.; ^2^Medical Governance Research Institute, Tokyo, Japan.; ^3^Faculty of Medicine, Comenius University, Bratislava, Slovakia.; ^4^Department of Gastroenterology, Sendai Kousei Hospital, Miyagi, Japan.; ^5^Kitasato University, Tokyo, Japan.

## Dear Editor,

The advent of novel therapeutics has caused the rising cost of drugs to become of increasing global concern. Countries with universal healthcare systems, such as Japan and the United Kingdom, face a seemingly unsolvable conundrum of how to sustain their exorbitantly expensive public healthcare systems in a cost-effective manner. In Japan, government policy is to reduce annual drug expenditure by 100 billion Japanese yen (US$913 million) in 2020. Japan is now a ‘super-aged’ society, meaning more than 28% of the population is aged 65 years and older. Elderly people need more medicine and, in view of the increasingly aging population and as part of “Abenomics,” the government is easing biomedical regulations and expediting approval of innovative drugs. However, it is difficult to identify whether patients or the pharmaceutical industry (Pharma) will be the main beneficiaries. What is clear, however, is that the economic burden of Japan’s universal national health insurance will rapidly become untenable.

Over the past decades, Japan’s revised drug regulation policy has accelerated the process for reviewing and approving novel pharmaceuticals and medical devices (PMD). However, as widely recognised and of concern internationally, Japan’s recent aim has seemingly been economic benefit for Pharma rather than health benefits for patients and, additionally, to attract clinical trials, expertise and relevant funding from both domestic sources as well as from overseas.^1-4^ With respect to regenerative medical products, the current regulatory framework, established under the 2013 Regenerative Medicine Promotion Act, is an illustrative example that has provoked significant international criticism.^1-4^ This regulation enabled Pharma to obtain a conditional, time-limited authorization (up to seven years) for novel regenerative products to be covered under Japan’s mandatory universal health insurance. These products must meet the following conditions: (1) cells are non-homogenous quality in nature; (2) clinical trials (other than confirmatory ones) must demonstrate “potential” efficacy; (3) products must not have any major adverse side-effects.^1^ While post-marketing surveillance and safety measures are required for approved products, a notable concern is that the legislative framework does not require an orthodox randomized, placebo-controlled Phase 3 trial. As of March 2020, conditional approval has been given to three regenerative medical products, HeartSheet^®^(human [autologous] skeletal myoblast-derived cell sheet), Stemirac^®^ (human [autologous] bone marrow-derived mesenchymal stem cell product), and Collategene^®^ (beperminogene perplasmid).^5^ Moreover, in late-2019, the PMD Act was revised to further promote approval of general PMD for life-threatening or rare diseases by expanding a conditional approval apparatus and by enacting the Sakigake Designation Scheme, which selectively enables accelerated approval of new medical products that are developed and produced in Japan.^6^

Grave concerns also now surround Japan’s drug regulatory processes. The nation has traditionally approved drugs several years behind the US Food and Drug Administration (FDA) and the European Medicines Agency (EMA).^7^ However, Japan is now approving an increasing number of products new to the global market. Worryingly, although the current review system and approval process is controlled by the PMD Agency and Japan’s Ministry of Health, Labour and Welfare, they are not of the same caliber as those of the FDA and EMA with regard to scientific standards. For example, in 2019, an oral FLT3-ITD inhibitor, quizartinib, Vanflyta^®^ supplied by the Daiichi Sankyo Company was approved in Japan, while the FDA and EMA refused approval due to substantive issues arising from a confirmatory Phase 3 trial of the drug.^8^

In Japan, virtually all PMD approved by the Minister of Health, Labour and Welfare are covered by health insurance, with very few exceptions. This differs from the process existing between the EMA and the UK’s National Health Service (NHS) and National Institute for Health and Care Excellence (NICE). For example, the EMA-approved atezolizumab, Tecentriq^®^, is not covered by the NHS’s Cancer Drugs Fund, as its cost-effectiveness did not meet NICE criteria as an appropriate use of NHS resources, even for end-of-life treatment.^9^ Perversely, in view of the goal of reducing drug prices and lowering the unbearable cost of ever-increasing national health insurance, in March 2020, Japan’s government approved the use of Novartis’ onasemnogene abeparvovec-xioi, Zolgensma^®^, which is believed to be the most expensive drug in the world. In the United States it costs over $2 million per treatment, while in Japan, the drug will be covered under the country’s universal national health insurance scheme.

It is a government’s responsibility to protect and advance the interests of the health of its citizens. However, Japan’s recent revisions of drug regulation policy and sub-optimal drug review quality mechanisms seem ill considered. They portend a potentially destructive extra burden for an already over-extended health insurance system, as well as risking benefits for patients. They must be revisited and revised, with the paramount goal being the promotion of health (for the many) rather than wealth (for a few).

## Ethical issues

Not applicable.

## Competing interests

AO and TT report personal fees from MNES Inc., and HS reports an honorarium from TAIHO Pharmaceutical Co., Ltd., outside of the submitted work. All other authors declare that they have no competing interests.

## Authors’ contributions

AO, YS, and AC wrote the manuscript. All authors contributed to conception and design of the study, and critical revision of the paper. All authors read and approved the final manuscript.

## Authors’ affiliations

^1^Department of Breast Surgery, Jyoban Hospital of Tokiwa Foundation, Fukushima, Japan. ^2^Medical Governance Research Institute, Tokyo, Japan. ^3^Faculty of Medicine, Comenius University, Bratislava, Slovakia. ^4^Department of Gastroenterology, Sendai Kousei Hospital, Miyagi, Japan. ^5^Kitasato University, Tokyo, Japan.
